# Novel silver nanoparticle-based biomaterials for combating *Klebsiella pneumoniae* biofilms

**DOI:** 10.3389/fmicb.2024.1507274

**Published:** 2025-01-09

**Authors:** Eslam Elashkar, Rihaf Alfaraj, Ola M. El-Borady, Mahmoud M. Amer, Abdelazeem M. Algammal, Azza S. El-Demerdash

**Affiliations:** ^1^Department of Botany and Microbiology, Faculty of Science, Benha University, Benha, Egypt; ^2^Department of Pharmaceutics, College of Pharmacy, King Saud University, Riyadh, Saudi Arabia; ^3^Institute of Nanoscience and Nanotechnology, Kafrelsheikh University, Kafr ElSheikh, Egypt; ^4^Department of Bacteriology, Immunology, and Mycology, Faculty of Veterinary Medicine, Suez Canal University, Ismailia, Egypt; ^5^Laboratory of Biotechnology, Department of Microbiology, Agricultural Research Center, Animal Health Research Institute, Zagazig, Egypt

**Keywords:** antibiofilm potential, chitosan, cytotoxicity, *Klebsiella pneumoniae*, silver nanoparticles, sodium fluoride

## Abstract

**Background:**

*Klebsiella pneumoniae* is a significant nosocomial pathogen that has developed resistance to multiple antibiotics, often forming biofilms that enhance its virulence. This study investigated the efficacy of a novel nanoformulation, AgNPs@chitosan-NaF, in combating *K. pneumoniae* biofilms.

**Methods:**

Antimicrobial susceptibility testing was performed to assess the antibiotic resistance profile of *K. pneumoniae* isolates. The antibiofilm activity of AgNPs@chitosan-NaF was evaluated using crystal violet staining and scanning electron microscopy. The underlying mechanisms of action were investigated through gene expression analysis.

**Results:**

The majority of *K. pneumoniae* isolates exhibited high levels of multidrug resistance. AgNPs@chitosan-NaF demonstrated superior biofilm inhibition compared to AgNPs@chitosan, significantly reducing biofilm biomass and disrupting biofilm architecture at MICs ranging from 0.125 to 1 μg/mL. Mechanistic studies revealed that the nanoformulation downregulated the expression of key biofilm-associated genes, including *treC*, *fimA*, *mrkA*, and *ecpA*. While AgNPs@chitosan-NaF exhibited a concentration-dependent cytotoxic effect on both normal and cancer cell lines, minimal cytotoxicity was observed at concentrations below 31.25 μg/mL.

**Conclusion:**

This study highlights the synergistic effect of silver nanoparticles, chitosan, and sodium fluoride in combating *K. pneumoniae* biofilms. The nanoformulation, AgNPs@chitosan-NaF, emerges as a promising therapeutic strategy to address the challenge of multidrug-resistant bacterial infections.

## Introduction

*Klebsiella pneumoniae*, a significant pathogen, poses a serious threat to global health due to its ability to form robust biofilms and develop multidrug resistance (Al Bshabshe et al., 2020). Biofilms, communities of bacteria embedded in a protective matrix, shield *K. pneumoniae* from antibiotics and other stressors, making infections difficult to treat. The emergence of multidrug-resistant *K. pneumoniae* strains has further complicated treatment options, contributing to high mortality rates, especially in vulnerable populations (El-Demerdash et al., 2018, 2023a; Guerra et al., 2022). Additionally, the biofilm-forming nature of *K. pneumoniae* can lead to persistent infections, requiring prolonged treatment and increasing the risk of complications (Abbas et al., 2024).

The development of innovative antimicrobial methods is essential to combat biofilm-forming bacteria, which often exhibit resistance to conventional antibiotics (Sharaf et al., 2022; El-Demerdash et al., 2023d; Ibrahim, 2023).

Nanoparticles, with their large surface area and tunable surface chemistry, offer a promising alternative to traditional antibiotics for combating multidrug-resistant (MDR) pathogens (Rudramurthy et al., 2016). Silver nanoparticles, in particular, have demonstrated potent antimicrobial activity and the ability to disrupt biofilm formation, a critical challenge in treating bacterial infections (Mba and Nweze, 2021; Saad et al., 2024). This has fueled increased research into nanoparticles as a potential solution to overcome antibiotic resistance (Franci et al., 2015; Abd El-Emam et al., 2024; Hashem et al., 2024).

Chitosan, a natural biopolymer, exhibits antimicrobial properties and biocompatibility, making it a promising candidate for biomedical applications. Chitosan nanoparticles, combining the advantages of chitosan with the unique properties of nanomaterials, have demonstrated potent antimicrobial activity against a wide range of microorganisms, including bacteria and fungi. Additionally, these nanoparticles have shown promise in combating biofilm formation, a critical challenge in treating infections caused by multidrug-resistant pathogens (El-Naggar et al., 2022; Ali et al., 2023; Desai et al., 2023).

This study aims to investigate the antibiofilm efficacy of silver nanoparticles encapsulated in chitosan (AgNPs@chitosan) and further enhanced with sodium fluoride (AgNPs@chitosan-NaF) against *Klebsiella pneumoniae* biofilms. We characterized the physicochemical properties of the nanocomposites, evaluate their antibiofilm activity using quantitative assays, and explored their mechanisms of action by examining biofilm architecture and gene expression. Additionally, we assessed the cytotoxicity of the nanocomposites against normal and cancer cell lines to evaluate their biocompatibility.

## Materials and methods

### Nano-materials

Silver nitrate (AgNO₃), sodium borohydride (NaBH₄), chitosan (medium molecular weight, 50–190 kDa, deacetylation degree 90.2%), and sodium fluoride (NaF) were purchased from Merck, Germany, and Fluka, Germany. Glacial acetic acid was obtained from ADWIC, Egypt. Double-distilled water was used to prepare all solutions. All chemicals were of analytical grade and used without further purification.

### Synthesis of AgNPs@chitosan and AgNPs@chitosan-NaF

- *Chitosan solution preparation*: 0.075 g of chitosan was dissolved in 200 mL of 2% acetic acid solution and stirred overnight at 600 rpm at room temperature.
- *AgNPs@chitosan synthesis*: 60 mL of the cooled chitosan solution was mixed with 4.0 mL of 0.012 M AgNO₃ solution for 15 min. Then, 0.3 mL of 0.8 M NaBH₄ was added dropwise to the mixture under constant stirring for 15 min, resulting in the formation of AgNPs@chitosan.
- *AgNPs@chitosan-NaF synthesis*: 0.1 g of NaF was added to the AgNPs@chitosan solution after the complete addition of NaBH₄.

### Nanoparticles characterization

- *Morphology and size*: Transmission electron microscopy (TEM) was used to examine the morphology and size distribution of the synthesized nanoparticles.
- *Zeta potential*: Zeta potential measurements were conducted to assess the surface charge of the nanoparticles.
- *Optical properties*: UV–Vis spectroscopy was employed to analyze the optical properties of the nanoparticles.
- *Functional groups*: Fourier-transform infrared (FTIR) spectroscopy was used to identify the functional groups present in the nanoparticles.
- *Crystalline structure*: X-ray diffraction (XRD) was used to determine the crystalline structure and phase purity of the nanoparticles.

### Determination of chitosan@AgNPs and chitosan@AgNPs-F nanoparticles cytotoxicity on normal and cancer lung cells

#### The MTT assay

A cell viability assay was performed using the MTT (3-(4,5-dimethylthiazol-2-yl)-2,5-diphenyltetrazolium bromide) method. To enhance cell adhesion, cells were seeded at a density of 1×10^5^ cells/mL in 96-well plates and then incubated for 24 h at 37°C with 5% CO2. After removing the culture medium, AgNPs@chitosan and AgNPs@chitosan-NaF nanoparticles were serially diluted in fresh medium and added to the cells. Control wells only received the culture medium. The plates were then incubated at 37°C for another 24 h.

Next, 20 μL of MTT solution (5 mg/mL in PBS) was added to each well followed by a 4-h incubation at 37°C. After dissolving the formazan crystals in 100 μL of DMSO, the absorbance was measured at 560 nm using a microplate reader Cell viability was calculated relative to the control group (Hao et al., 2013).

#### Cellular morphology assessment

Cells were observed under an inverted microscope after 48 h of exposure to different concentrations of AgNPs@chitosan and AgNPs@chitosan-NaF or the control. The morphology of normal WI-38 and A549 ATCC-185 cancer cell lines was examined for any changes, and images were captured.

### Sample collection

A total of 57 clinical specimens (19 blood, 19 urine, and 19 sputum samples) were collected from patients at Benha Fever Hospital in Beha City, Egypt. Additionally, 32 mastitic milk samples from various farms and 31 minced meat samples from supermarkets in Sharkia governorate were obtained. All samples were collected under strict hygienic conditions.

### Isolation, identification, and molecular confirmation of *Klebsiella pneumoniae*

*Klebsiella* species were isolated from clinical and environmental samples using selective MacConkey agar. Pink, mucoid colonies were further purified and subjected to Gram staining and biochemical tests, including TSI, oxidase, indole, urease, and motility tests, for presumptive identification (Garcia, 2010).

*Klebsiella pneumoniae* was confirmed through PCR amplification of the 16S rRNA gene. Genomic DNA was extracted from bacterial isolates using the QIAamp DNA Mini Kit. PCR amplification was performed using specific primers targeting the 16S rRNA gene (Table 1). A positive control strain (*Klebsiella pneumoniae* ATCC 13883) and a negative control (no template) were included in each PCR run.

**Table 1:** Primers sequences and target genes of *Klebsiella* biofilm genes for Syper green RT-PCR.

Target gene	Primers sequences	Reference
*16S rRNA*	ATT TGA AGA GGT TGC AAA CGA T	Turton et al. (2010)
	TTC ACT CTG AAG TTT TCT TGT GTT C	
*treC*	CCGACAGCGGGCAGTATT	Wu et al. (2011)
	CGCCGGATTCTCCCAGTT	
*fimA*	CGGACGGTACGCTGTATTTT	Alcántar-Curiel et al. (2013)
	GCTTCGGCGTTGTCTTTATC	
*mrkA*	CGGTAAAGTTACCGACGTATCTTGTACTG	Alcántar-Curiel et al. (2013)
	GCTGTTAACCACACCGGTGGTAAC	
*ecpA*	GCAACAGCCAAAAAAGACACC	Alcántar-Curiel et al. (2013)
	CCAGGTCGCGTCGAACTG	

### Quantification of biofilm formation

Biofilm formation was quantified using the crystal violet staining method (Stepanović et al., 2000). Bacterial suspensions were cultured in 96-well microtiter plates for 24 h. After removing the planktonic cells, adherent biofilms were stained with crystal violet and the bound dye was solubilized with ethanol-acetone. The absorbance of the solubilized dye at 595 nm was measured to determine biofilm biomass. Isolates were classified as strong, moderate, weak, or non-biofilm producers based on their absorbance values relative to a positive control strain.

### Antimicrobial susceptibility test

#### Disk diffusion method

In accordance with the Clinical and Laboratory Standards Institute (CLSI) recommendations, antimicrobial susceptibility testing was conducted using the Kirby-Bauer disk diffusion method (Bauer et al., 1966). Muller-Hinton agar (MHA) served as the growth medium. Antimicrobial disks were placed on MHA plates after standardized bacterial suspensions were inoculated. The following antibiotic disks were used: ampicillin (AM, 10 μg), amoxicillin (AX, 25 μg), amoxicillin-clavulanic acid (AMC, 30 μg), amikacin (AK, 30 μg), azithromycin (AZM, 15 μg), aztreonam (ATM, 10 μg), clindamycin (DA, 2 μg), cefodroxil (CFR, 30 μg), chloramphenicol (C, 30 μg), ceftriaxone (CRO, 30 μg), cefoxitin (FOX, 30 μg), ciprofloxacin (CIP, 5 μg), cefaclor (CEC, 30 μg), ceftazidime (CAZ, 30 μg), doxycycline (DO, 30 μg), erythromycin (E, 15 μg), gatifloxacin (GAT, 5 μg), gentamicin (CN, 10 μg), levofloxacin (LEV, 5 μg), nalidixic acid (NA, 30 μg), nitrofurantoin (F, 300 μg), norfloxacin (NOR, 10 μg), ofloxacin (OFX, 5 μg), penicillin (P, 10 μg), sulbactam/ampicillin (SAM, 20 μg), tetracycline (TE, 30 μg), trimethoprim/sulfamethoxazole (SXT, 25 μg), and tobramycin (TOB, 10 μg) obtained from Bioclone (Turkey) and Oxoid (England). Plates were incubated at 37°C for 18–24 h, and inhibition zone diameters were measured to determine antimicrobial susceptibility (CLSI, 2020). Multidrug-resistant isolates were defined as those exhibiting resistance to three or more distinct classes of antibiotics (MDR) and categorized with the recommendation of Tambekar et al. (2006).

#### Agar well diffusion method

To evaluate the antibacterial effectiveness of the synthetic nano particles. We utilized the agar well diffusion method (Venkataraju et al., 2014). A consistent suspension of *Klebsiella pneumoniae* was evenly spread on nutrient agar plates. Wells were created in the agar using aseptic techniques, and a predetermined volume of the nanoparticle suspension was added to each well. Control wells containing only the solvent were also included. The plates were incubated aerobically at 37°C for 24 h. The antibacterial potency of the nanoparticles was determined by measuring the diameter of the inhibition zones surrounding the wells.

#### Determination of minimum inhibitory concentration

Using the broth microdilution method, we determined the MIC values for both the synthesized nanoparticles and antibiotics according to the criteria outlined by (CLSI, 2020). We created a series of two-fold dilutions of the test compounds in a 96-well microtiter plate, with the highest concentration being 1,024 μg/mL. An overnight culture of *Klebsiella pneumoniae* was adjusted to a final concentration of approximately 5 × 10^5^ colony-forming units (CFU)/mL. A volume of 100 μL of the bacterial suspension was added to each well containing the test compounds. The plates were incubated at 37°C for 24 h. The MIC was defined as the lowest concentration of the test compound that inhibited visible bacterial growth.

### Transmission electron microscopy

Analysis to visualize the morphological changes in *Klebsiella pneumoniae* biofilms induced by antibiotic treatment and nanoparticle exposure, transmission electron microscopy (TEM) was employed. Biofilm samples were collected and processed for TEM analysis according to standard protocols. Briefly, biofilms were fixed with glutaraldehyde, dehydrated, embedded in resin, and sectioned for imaging. TEM analysis was performed using a JEOL-JSM-5500LV microscope operated at an acceleration voltage of 80 kV.

### Biofilm genes expression analysis

Total RNA was extracted from bacterial cultures using the QIAamp RNeasy Mini Kit and quantified using a NanoDrop spectrophotometer. RNA purity was assessed by calculating the A260/A280 and A260/A230 ratios, where values of 1.8–2.0 and 2.0–2.2, respectively, indicate high-quality RNA. qPCR analysis was performed using SYBR Green chemistry on a StepOnePlus Real-Time PCR System utilizing primers listed in Table 1. A 20 μL reaction mixture containing 10 μL of 2x HERA SYBR® Green RT-qPCR Master Mix (Willowfort, United Kingdom), 1 μL of RT Enzyme Mix (20X), 1 μL of each primer (20 pmol), 3 μL of nuclease-free water, and 5 μL of RNA template was prepared. The program included an initial denaturation step at 94°C for 15 min, followed by 40 cycles of denaturation (94°C for15s), annealing (55°C for 30s), and extension (72°C for 30s). A final extension step at 72°C for 10 min concluded the reaction. Relative gene expression levels were calculated using the 2^-ΔΔCt^ method, with the 16S rRNA gene serving as a reference gene (Yuan et al., 2006).

### Statistical analysis

Data were edited in MS Excel (Microsoft Corporation, Redmond, WA, United States). The Levene and Shapiro–Wilk tests were conducted in order to check for normality and homogeneity of variance (Razali and Wah, 2011). One way- Anova of statistical analysis system (SAS, 2012) was used for assessing gene transcription of *treC*, *fimA*, *mrkA*, and *espA*. Multiple comparisons among means were carried out by the Duncan’s Multiple Range Test (Steel and Torrie, 1980). Results of antimicrobial resistant were examined by fisher exact test. Results were expressed as means ± SE. A logistic regression model (PROC LOGISTIC;) was run with the level of significance set at *α* = 0.05 to examine the effects of potential risk factors, including clinical samples, sex, age, sample sources, and sample type on the prevalence of *Klebsiella*. Statistical significance was accepted at probability less than 0.05.

## Results

### UV–vis spectroscopy

**Figure 1:**
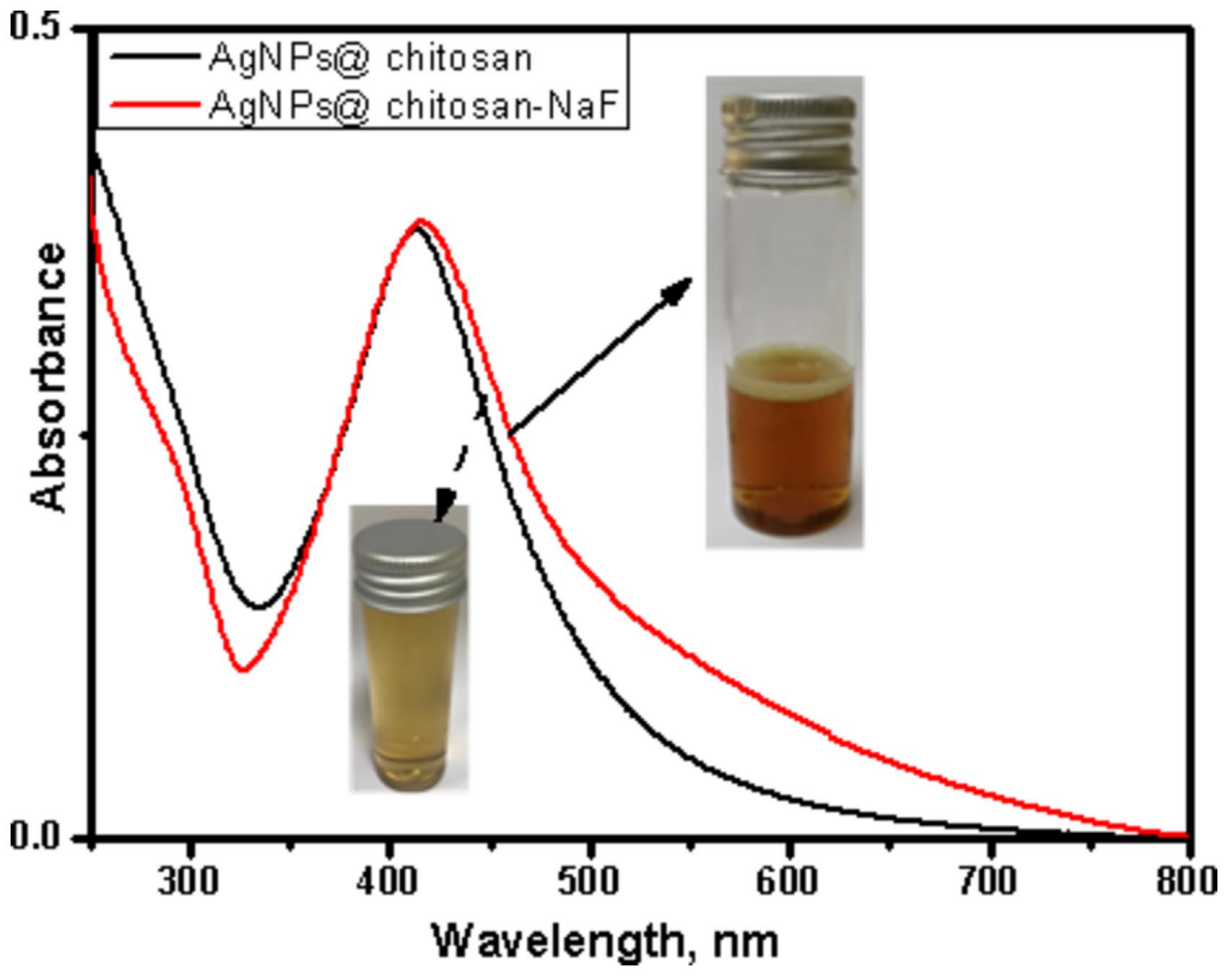
UV–Vis absorption spectra of AgNPs@chitosan and AgNPs@chitosan-NaF, and the inset is their photo images.

UV–Vis absorption spectra of AgNPs@chitosan and AgNPs@chitosan-NaF exhibited characteristic surface plasmon resonance (SPR) peaks centered at approximately 413 nm and 415 nm, respectively (Figure 1). The appearance of these SPR peaks confirmed the formation of AgNPs within the chitosan matrix. TEM images of the two synthesized AgNPs samples clearly show the formation of uniform, well-defined particles, confirming their characteristic SPR peaks.

### High-resolution transmission electron microscope imaging

**Figure 2:**
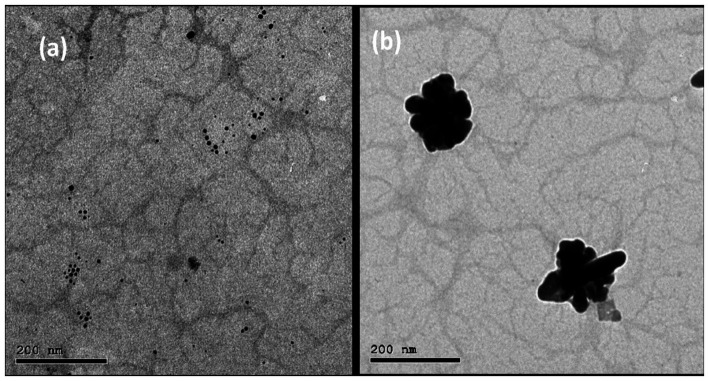
**(A)** The TEM image of the synthesized AgNPs@ chitosan and **(B)** AgNPs@ chitosan-NaF.

The morphological characteristics of the two synthesized nanocomposites were investigated using HR-TEM. Figure 2A demonstrates a representative TEM image of AgNPs@chitosan that appeared as agglomerated small particles. These agglomerates look like flowers in the range of 100 nm. Additionally, the TEM images of AgNPs@chitosan-NaF signify the formation of small spherical AgNPs dispersed over a chitosan matrix (Figure 2B).

### Energy-dispersive X-ray spectroscopy analysis

**Figure 3:**
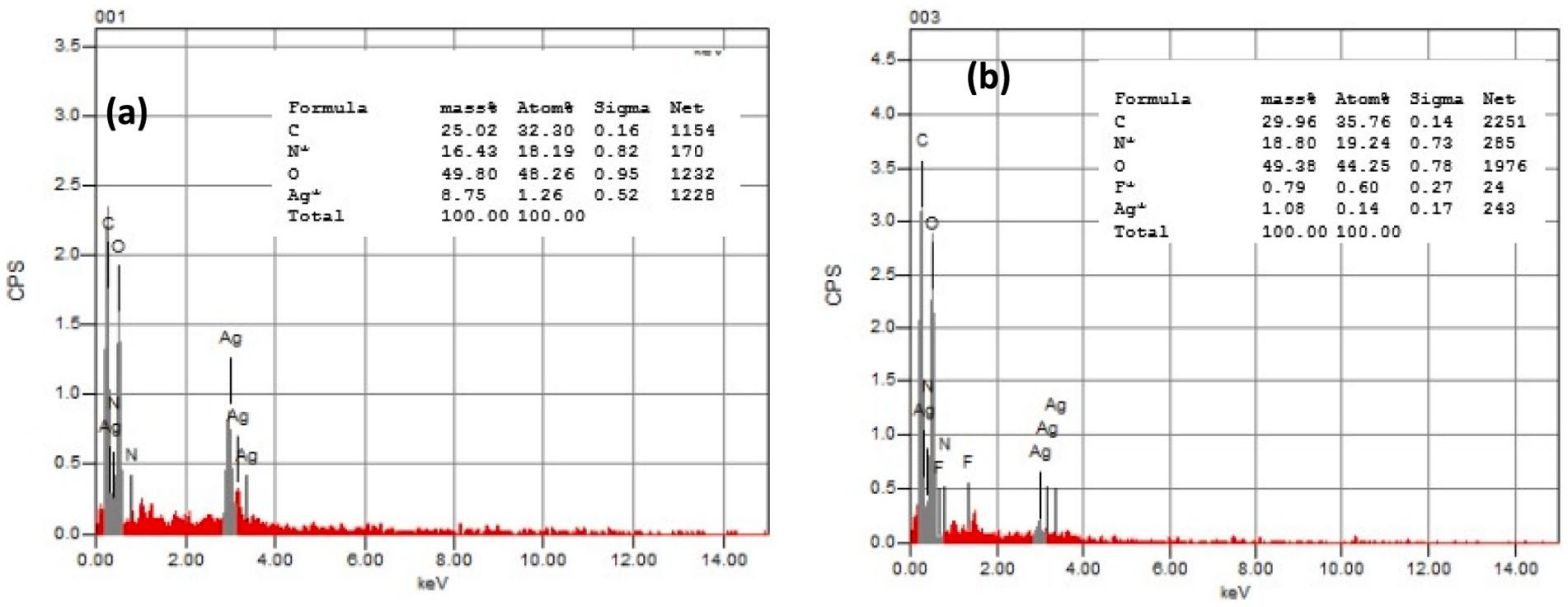
The EDX pattern of **(A)** the synthesized AgNPs@chitosan and **(B)** AgNPs@chitosan-NaF.

The elemental composition of AgNPs@chitosan and AgNPs@chitosan-NaF was determined using EDX analysis. The EDX spectrum of AgNPs@chitosan revealed the presence of carbon (25%), oxygen (49.5%), nitrogen (16.42%), and silver (8.75%) (Figure 3A). For AgNPs@chitosan-NaF, the EDX spectrum showed peaks corresponding to carbon (29.96%), oxygen (49.35%), nitrogen (1.09%), silver (2.1%), and fluorine (0.79%) (Figure 3B).

### FTIR spectroscopy

**Figure 4:**
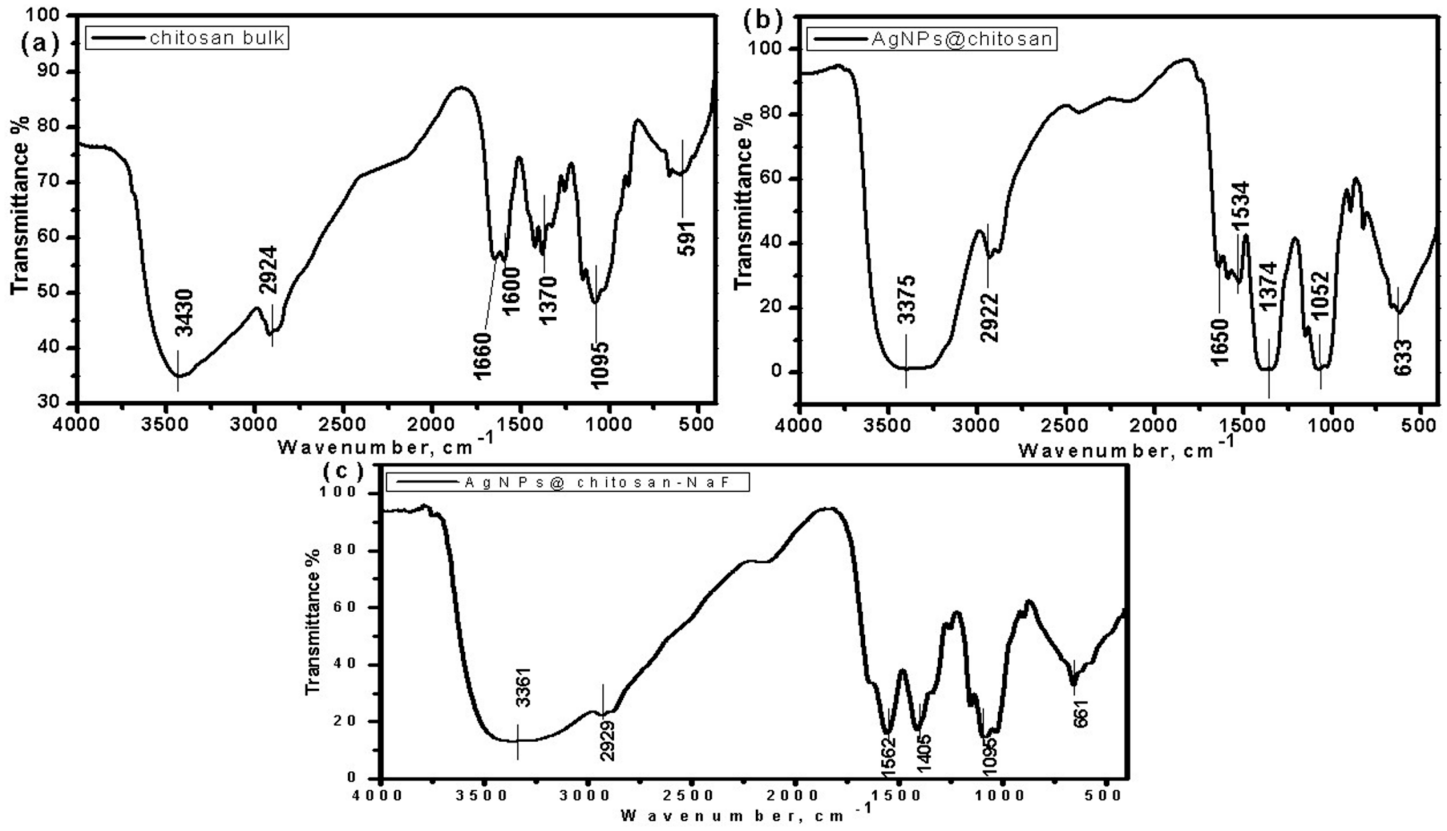
The FTIR spectra of **(A)** chitosan bulk, **(B)** AgNPs@chitosan and **(C)** AgNPs@chitosan-NaF.

Fourier-transform infrared (FTIR) spectroscopy was employed to investigate the chemical interactions between chitosan and AgNPs in the synthesized nanocomposites. The FTIR spectra of chitosan, AgNPs@chitosan, and AgNPs@chitosan-NaF are shown in Figures 4A–C. Compared to pure chitosan, the spectra of the nanocomposites exhibited shifts in the characteristic peaks corresponding to the O-H, N-H, and C-O-C stretching vibrations. A new peak at approximately 661 cm^−1^ emerged in the spectra of the nanocomposites (Table 2).

**Table 2:** The FTIR analysis of chitosan bulk, AgNPs@chitosan and AgNPs@chitosan-NaF.

Functional group	Chitosan bulk	AgNPs@chitosan	AgNPs@chitosan-NaF
OH or NH-amine in amino groups	3,430 cm^−1^	3,375 cm^−1^, sharp	3,361 cm^−^, sharp
C–H and C–N stretching	2,924 cm^−1^	2,922 cm^−1^	2,929 cm^−1^
N–H bending group of amides	1,660 cm^−1^	Split into three peaks, 1,650 cm^−1^	Sharp, 1,562 cm^−1^
N–H angular deformation in CO NH_2_ plane	1,600 cm^−1^	1,534 cm^−1^	1,562 cm^−1^
Protein stretch carbonyl C–O–C Stretching	1,095 cm^−1^	1,052 cm^−1^, sharp	1,095 cm^−1^, very sharp
Organic–metal bond	591 cm^−1^	661 cm^−1^	661 cm^−1^

### X-ray diffractions analysis

**Figure 5:**
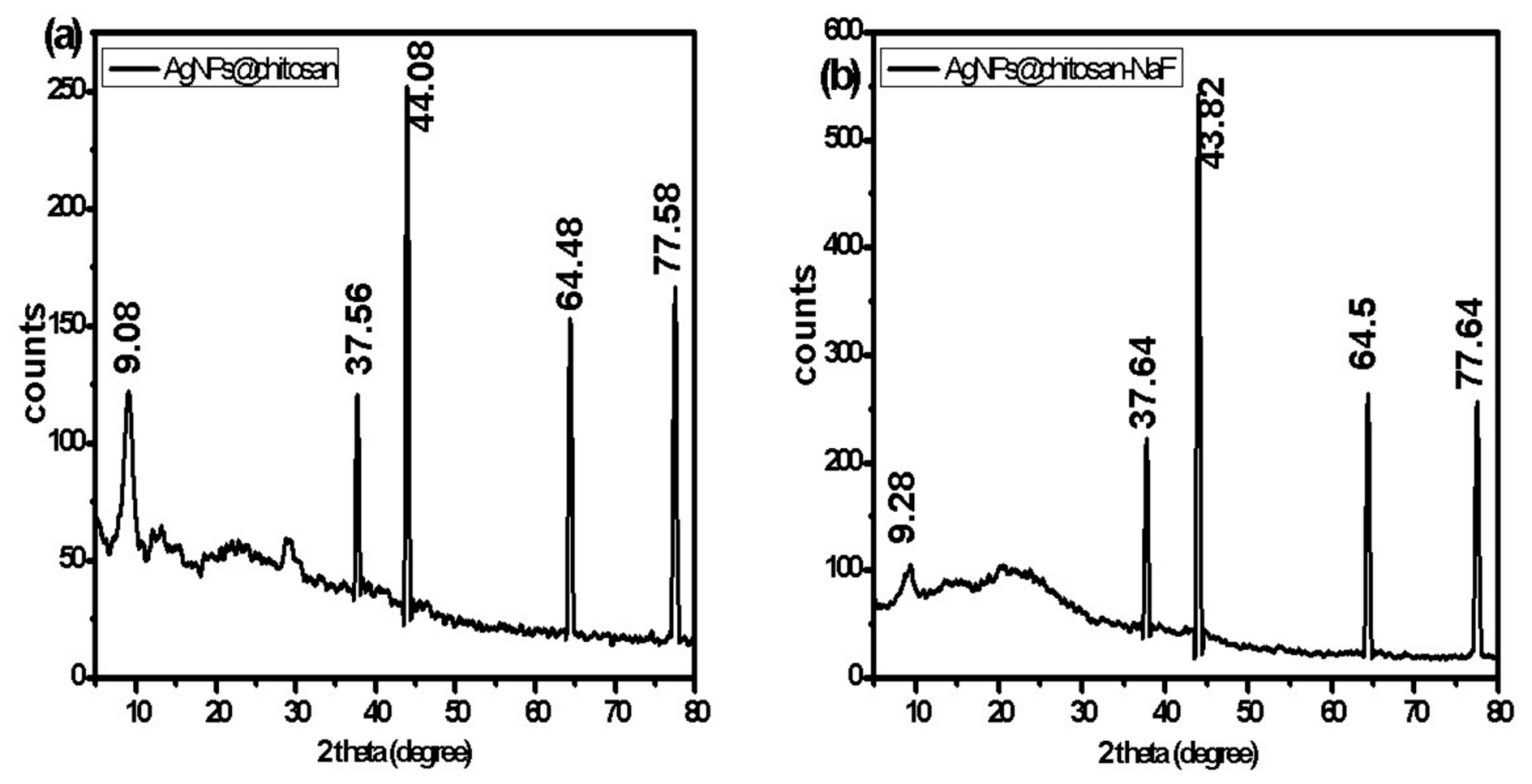
The XRD patterns of **(A)** AgNPs@chitosan and **(B)** AgNPs@chitosan-NaF.

The crystalline structure of AgNPs@chitosan and AgNPs@chitosan-NaF was investigated using XRD analysis. The XRD patterns exhibited characteristic diffraction peaks at approximately 38°, 44°, 64°, and 77° 2θ, corresponding to the (111), (200), (220), and (311) planes of face-centered cubic (fcc) silver, respectively (Figures 5A,B). Broad diffraction peaks in the range of 10–25° 2θ were attributed to the amorphous nature of chitosan.

### Cell viability and cytotoxicity

**Figure 6:**
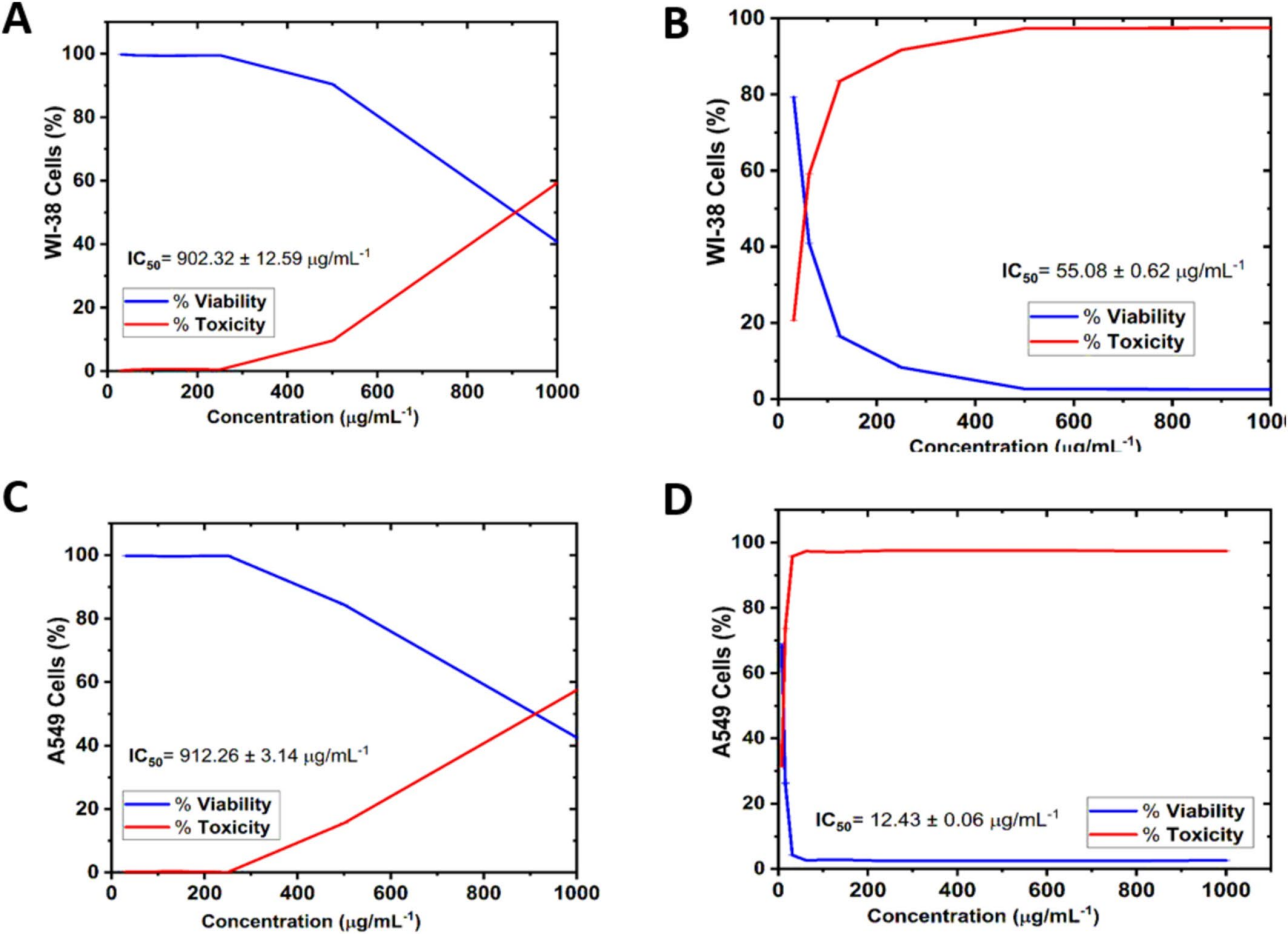
Cell viability and Cytotoxicity of AgNPs@chitosan and AgNPs@chitosan-NaF on normal lung fibroblast WI-38 cells **(A,B)**, and lung carcinoma epithelial A549 ATCC-185 cells **(C,D)** for 24 h. The results were taken from replicated (*n* = 3) (Mean ± SD).

The cytotoxicity of AgNPs@chitosan and AgNPs@chitosan-NaF was calculated based on the growth inhibition compared to the control group. AgNPs@chitosan and AgNPs@chitosan-NaF were tested against WI-38 were isolated from human fetal normal lung fibroblast cells, (Figures 6A,B), and A549 ATCC-185 were isolated from lung carcinoma epithelial cells (Figures 6C,D), at concentrations of 7.81 to 1,000 μg mL^−1^ to determine their ability to inhibit normal and cancer cell growth. The test was conducted for 24 h at 37°C. For each concentration, there were three duplicates in addition to the untreated control sample. The rate of growth inhibition for AgNPs@chitosan and AgNPs@chitosan-NaF relative to the control group, which grew at 100%, was used to calculate the severity of the toxicological impact.

For cell viability, AgNPs@chitosan and AgNPs@chitosan-NaF samples at 31.25 and 1,000 μg mL^−1^ caused significant changes in normal WI-38 cells in a concentration-dependent manner compared with the control. Where, Figures 6A,B show that the WI-38 cells treated with AgNPs@chitosan-NaF exhibited clear cell destruction and were more impacted than those treated with AgNPs@chitosan. AgNPs@chitosan-NaF showed the morphological characteristics of apoptosis in a concentration-dependent manner at 31.25 to 62.5 μg mL^−1^. While, the cells treated with AgNPs@chitosan showed less uniformity, with membrane integrity loss, rounding, and shrinking, but remained intact at AgNPs@chitosan concentrations of 500 and 1,000 μg mL^−1^.

Overall, all the tested concentrations showed cytocompatibility at concentrations of up to at least 500 μg mL–1 (>90.42%) of AgNPs@chitosan, and 31.25 μg mL^−1^ (>79.28%) of AgNPs@chitosan-NaF after 24 h of incubation onto WI-38 cells, additionally, up to at least 500 μg mL^−1^ (>84.39%) of AgNPs@chitosan, and 7. 81 μg mL^−1^ (>68.47%) of AgNPs@chitosan-NaF after 24 h of incubation onto A549 ATCC-185 cells (Figures 6C,D).

For cytotoxicity of AgNPs@chitosan and AgNPs@chitosan-NaF against of normal lung fibroblast WI-38 cells, and of lung carcinoma epithelial A549 cells was dose-dependent at an inhibition concentration of 50% (IC_50_). The cytotoxicity values (IC_50_ values) were 902.32 ± 12.59 μg mL^−1^, and 55.08 ± 0.62 μg mL^−1^ for the AgNPs@chitosan and AgNPs@chitosan-NaF, respectively against WI-38 cells. While, IC_50_ values were 912.26 ± 3.14 μg mL^−1^, and 12.43 ± 0.06 μg mL^−1^ for the AgNPs@chitosan and AgNPs@chitosan-NaF, respectively, against A549 ATCC-185 cells. Further evaluation of the AgNPs@chitosan and AgNPs@chitosan-NaF showed the lowest level of cytotoxic activity against WI-38 cells against WI-38 cells at a concentration of less than 1,000 μg mL^−1^ (>59.25%),and at a concentration of less than 62.5 μg mL^−1^ (>59.10%), respectively (Figures 6A,B), and cytotoxic activity against A549 ATCC-185 cells at a concentration of less than 1,000 μg mL^−1^ (>57.45%),and at a concentration of less than 15.62 μg mL^−1^ (>73.67%), respectively (Figures 6C,D).

### Morphological features

**Figure 7:**
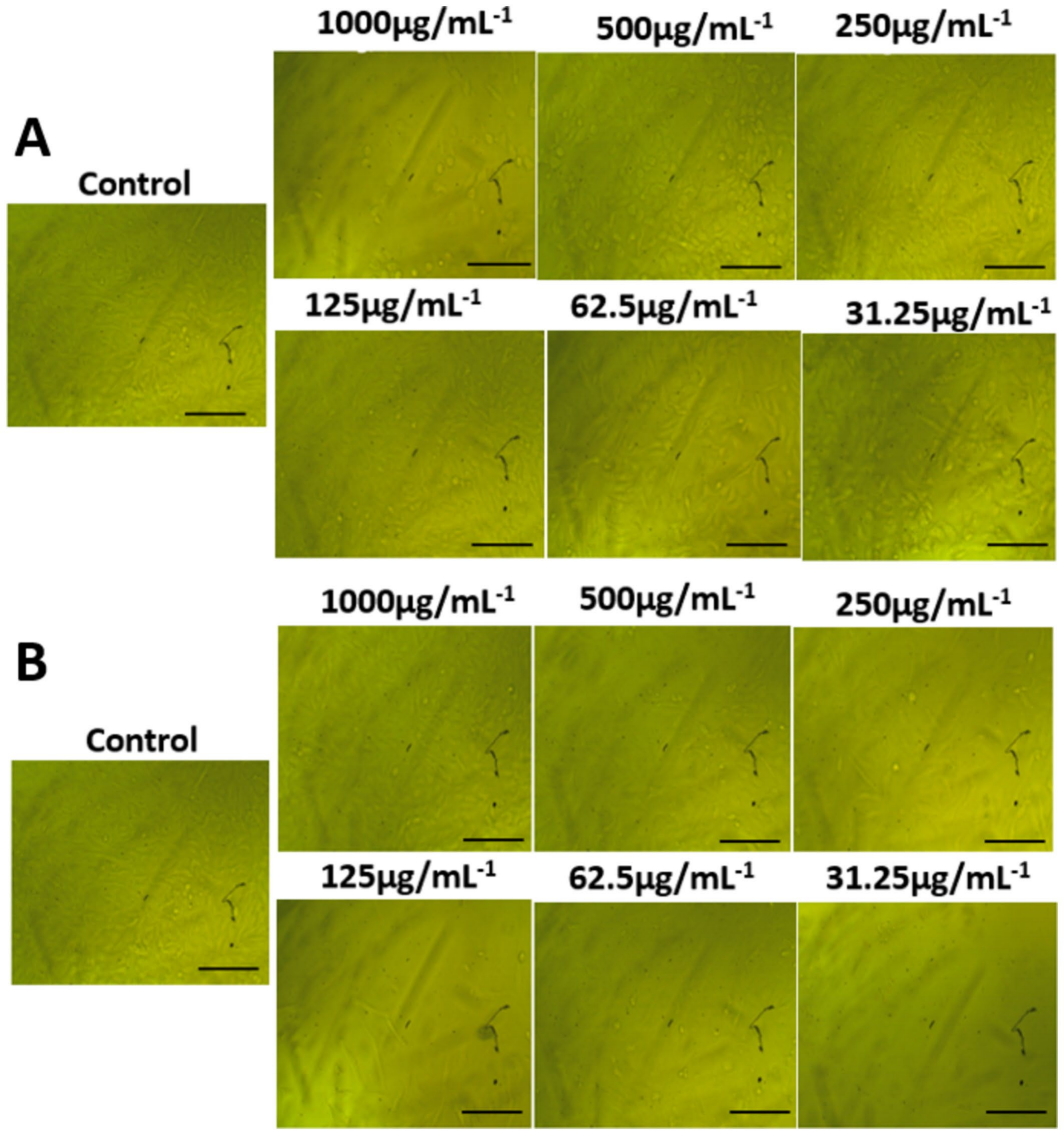
Morphological features on normal lung fibroblast WI-38 cells of **(A)** AgNPs@chitosan and **(B)** AgNPs@chitosan-NaF, the images were taken from the cells were treated with an average size of 10 nm for 24 h.

**Figure 8:**
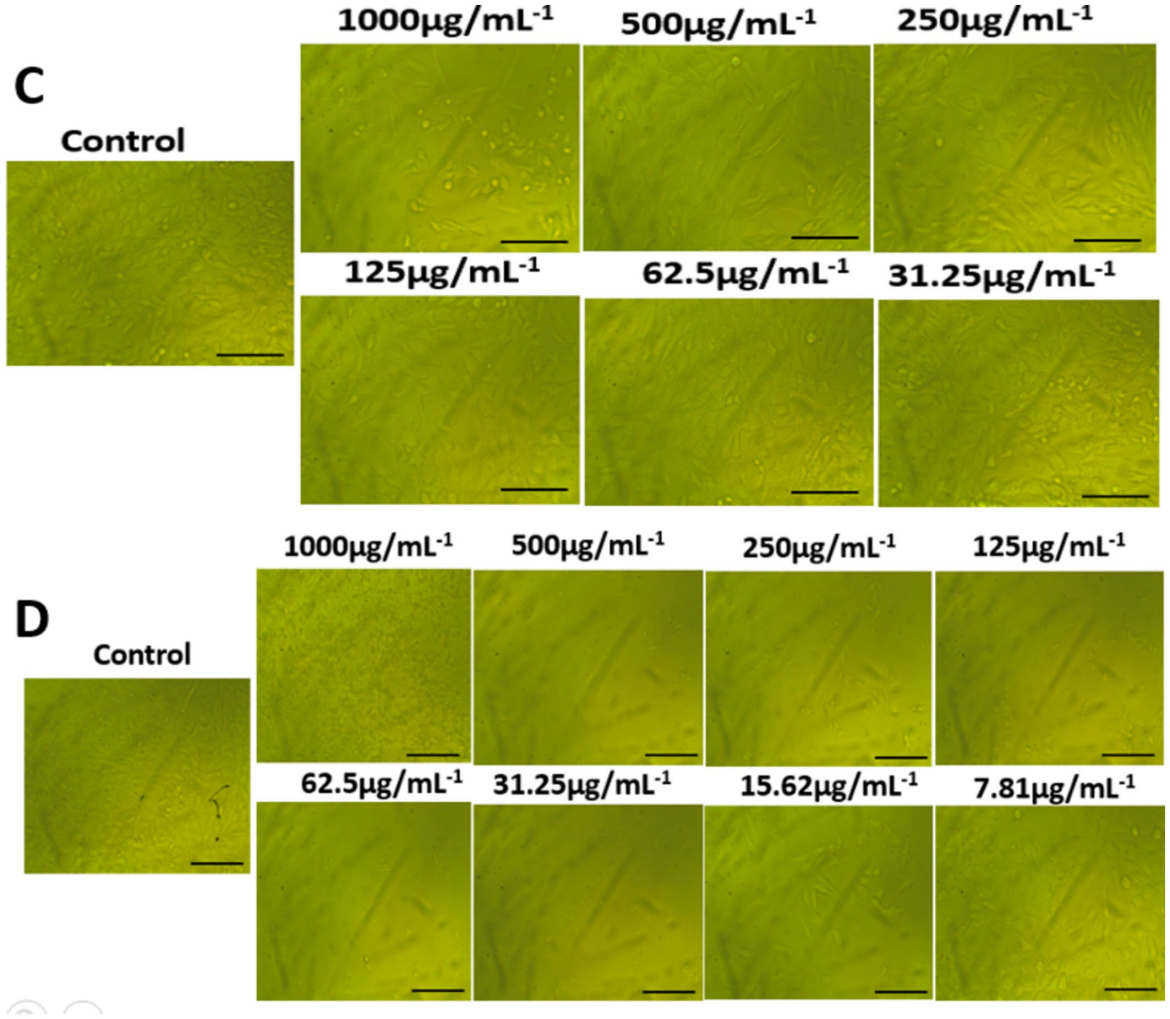
Morphological features on lung carcinoma epithelial A549 cells of **(A)** AgNPs@chitosan and **(B)** AgNPs@chitosan-NaF, the images were taken from the cells were treated with an average size of 10 nm for 24 h.

The morphological characteristics of untreated cells, as well as normal WI-38 and A549 ATCC-185 cancer cell lines treated with different amounts of AgNPs@chitosan and AgNPs@chitosan-NaF were compared (Figures 7, 8). Absorbance values obtained after capturing the red dye a long with the appropriate concentrations of AgNPs@chitosan and AgNPs@chitosan-NaF used in the viability studies, were analyzed using the 3 T3 Phototox program to assess the concentrations of the produced samples in various cell lines.

### Prevalence rate of *Klebsiella* spp

The prevalence of *K. pneumoniae* was determined to be 12.5% (15/120) based on genotypic identification using 16S rRNA gene-specific primer. Amplicon size analysis confirmed the identity of *K. pneumoniae* isolates, with a characteristic 130 bp band observed.

### Risk factors associated with *Klebsiella* prevalence

Logistic regression analysis identified several factors associated with the prevalence of *Klebsiella*. Clinical source was a significant predictor, with blood and sputum samples showing a lower risk compared to urine samples. Females were more likely to be colonized with *Klebsiella* than males. Age group analysis revealed a decreased risk of *Klebsiella* colonization in individuals aged 31–40 years and ≤ 41 years compared to the 20–30 age group. Additionally, human-derived samples exhibited a higher risk of *Klebsiella* colonization compared to animal and food samples, with minced meat samples showing the highest risk (Table 3).

**Table 3:** Logistic regression analysis of different risk factors associated with the prevalence of *Klebsiella.*

Item^1^	*n*	Positive	Negative	*β*	OR
Clinical sources					
Urine	8 (5/3)	5	3		Ref.
Blood	8 (4/4)	4	4	−0.5108	0.6
Sputum	2 (1/1)	1	1	−0.5108	0.6
Sex					
Male	2 (1/1)	4	4		Ref.
Female	10 (6/4)	6	4	0.4055	1.5
Age					
20–30	4 (3/1)	3	1		Ref.
31–40	7 (3/4)	3	4	−1.386	0.25*
≥41	7 (4/3)	4	3	−0.444	0.44*
Source					
Animal	16 (5/11)	5	11		Ref.
Human	18 (10/8)	10	8	1.0115	2.93*
Type					
Mastitis milk	9 (4/5)	4	5		Ref.
Minced meat	7 (6/1)	6	1	2.0148	7.5***

^1^β, regression coefficient; OR, odds ratio; CI, confidence interval (95%); and Ref.: reference. *p < 0.05; **p < 0.01; ***p < 0.001.

### Biofilm findings

Biofilm quantification analyses showed that 100% of the isolates were biofilm producers. The obtained isolates of this study had the following results for the categories of biofilm production: 25% weakly adherent, 37.5% moderately adherent, and 37.5% strongly adherent.

### Antibiotics susceptibility patterns

The antimicrobial susceptibility profiles of *Klebsiella* isolates from human and animal sources exhibited significant differences (*p* < 0.001). Isolates from animal sources demonstrated higher overall resistance rates compared to human isolates. Notably, all animal isolates were susceptible to chloramphenicol, while a high resistance rate was observed for doxycycline in mastitis milk samples. In contrast, human isolates exhibited a wider range of antibiotic resistance, with the highest resistance rates observed for chloramphenicol, gentamicin, and ceftriaxone.

**Figure 9:**
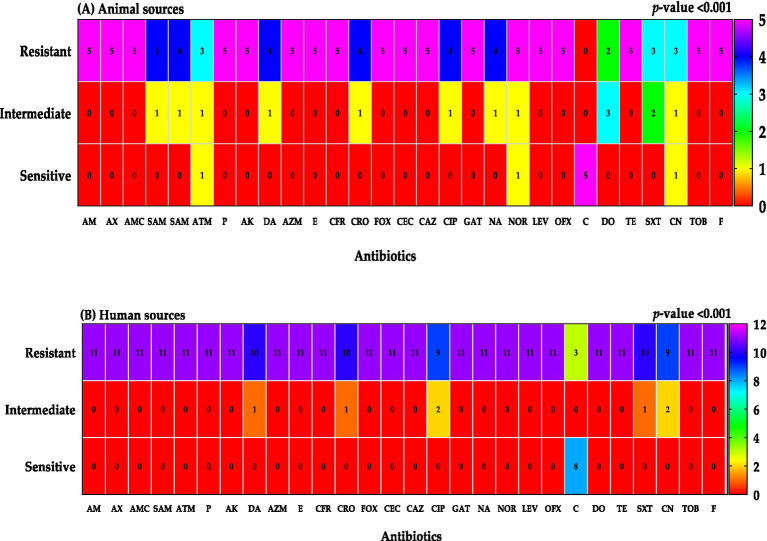
Resistant classifications to antibiotics were categorized by sample sources; animal sources **(A)** and human sources **(B)**.

**Figure 10:**
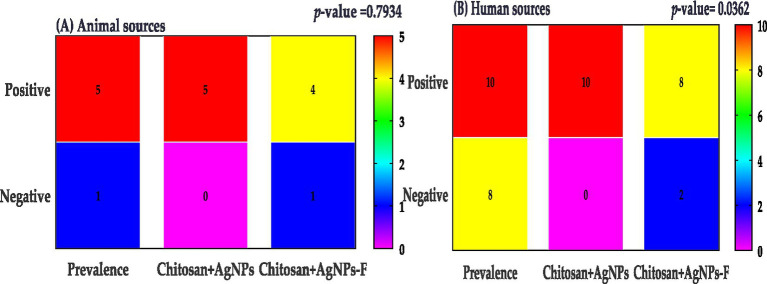
*Klebsiella* prevalence were categorized by sample sources; animal sources **(A)** and human sources **(B)**.

Analysis of antimicrobial resistance patterns based on clinical source, gender, and age revealed significant variations. Urine and blood samples exhibited higher resistance rates compared to sputum samples. Females demonstrated higher resistance rates than males, particularly for chloramphenicol. Older age groups showed higher resistance rates compared to younger age groups (Figures 8–10; Table 3).

### Nanoparticles activity

The AgNPs@chitosan and AgNPs@chitosan-NaF nanocomposites demonstrated potent antibacterial activity against *Klebsiella pneumoniae* isolates. This was evidenced by the formation of inhibition zones ranging from 20 to 35 mm in the agar diffusion assay and MICs ranging from 0.125 to 1 μg/mL.

### Isolated biofilm forming *Klebsiella* and the effect of the two silver chitosan nanoparticles on the biofilm using transmission electron microscope

**Figure 11:**
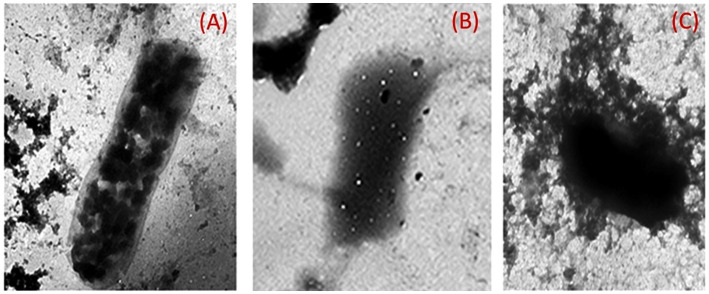
Transmission electron micrographs of *K. pneumoniae* cells; **(A)** Untreated *K. pneumoniae* cells; **(B)** *K. pneumoniae* cells treated with AgNPs@chitosan and **(C)** *K. pneumoniae* cells treated with AgNPs@chitosan-NaF.

The provided electro-micrograph images depicted the ultrastructural morphology of *K. pneumoniae* biofilm, both untreated and treated with chitosan-silver nanoparticle composites. The dark regions in the images represent the bacterial cells and extracellular matrix of the biofilm, while the lighter areas may indicate voids or spaces within the biofilm structure (Figure 11). The image of the untreated *Klebsiella pneumoniae* biofilm likely shows a dense, interconnected network of bacterial cells embedded in a matrix of extracellular polymeric substances (EPS). This matrix provides protection for the bacteria and contributes to the biofilm’s resistance to antibiotics and other stresses. The images of the biofilm treated with Chitosan@AgNPs-F show the profound disruptions in the biofilm structure, such as decreased bacterial density, increased porosity, or detachment of bacterial cells. These changes could be indicative of the antibiofilm activity of the treatment.

### Modulatory effect of AgNPs@chitosan and AgNPs@chitosan-NaF on biofilm of *Klebsiella pneumoniae* genotypically

**Figure 12:**
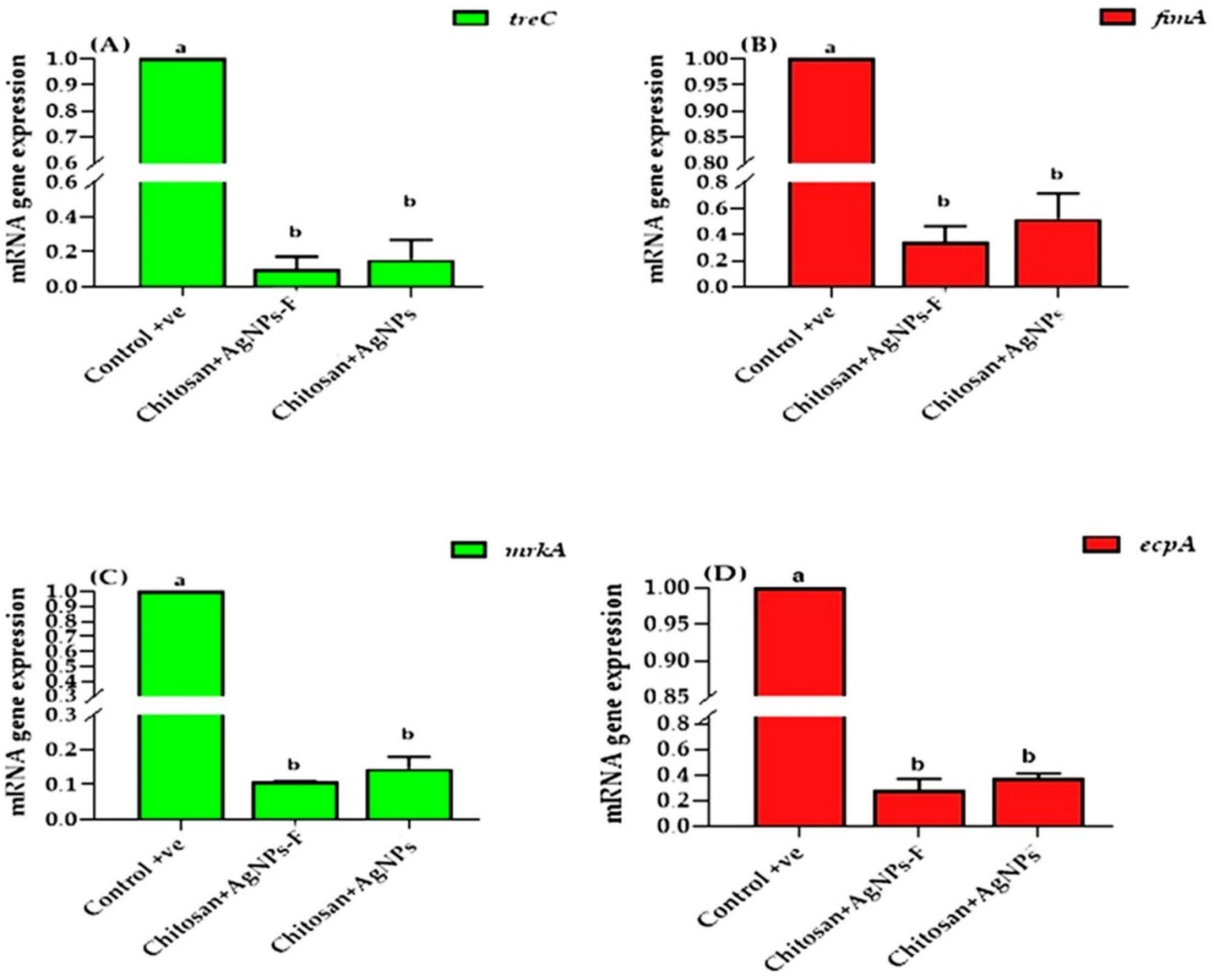
The effects of two tested silver nanoparticle-based biomaterial treatments on the expression of key biofilm genes in *K. pneumoniae*: *treC* **(A)**, *fimA* **(B)**, *mrkA* **(C)**, and *espA* **(D)**. The data is presented as means ± SEM.

The present results clearly indicated that the transcription of *treC, fimA, mrkA,* and *espA* was significantly down regulated in both two treated group compared to the control group, minimized in the group received chitosan+AgNPs-F (*p* < 0.05; Figure 12).

## Discussion

The UV–Vis absorption spectra findings are consistent with previous studies reporting the SPR band of AgNPs in the range of 400–420 nm. As reported by Mehr et al. (2015) well-defined silver nanoparticles (AgNPs) exhibit a surface plasmon resonance (SPR) peak at wavelengths less than 420 nm. The presence of a clear SPR peak in the UV–Vis spectra indicates the successful synthesis of AgNPs within the chitosan matrix.

The change observed in the AgNPs shape in both samples is tentatively attributed to the presence of NaF, which acts as an additional capping agent besides NaBH4. Consequently, the particle’s shape changed from a flowered-like shape into well-dispersed small spherical particles. This finding is consistent with previous reports by Larm et al. (2019) who confirmed the instability of NaBH4-capped AgNPs using TEM imaging.

The presence of fluorine in the AgNPs@chitosan-NaF nanocomposite is particularly significant as it suggests that NaF has been successfully incorporated into the chitosan matrix. This could potentially enhance the properties of the nanocomposite, such as its antimicrobial activity or biocompatibility.

The shifts in the characteristic peaks and the emergence of a new peak at 661 cm^−1^ in the FTIR spectra of the nanocomposites indicate the formation of chemical interactions between chitosan functional groups and AgNPs. This suggests that the AgNPs are successfully incorporated within the chitosan matrix, which could enhance the stability and properties of the nanocomposite. The presence of Ag-O bonds, as indicated by the new peak at 661 cm^−1^, further supports the formation of AgNPs within the chitosan matrix (Pawar et al., 2016).

The XRD results confirmed the formation of crystalline silver nanoparticles within the chitosan matrix, consistent with previous findings by Kaur et al. (2013). The presence of both silver and chitosan peaks in the XRD patterns indicated the successful formation of the AgNPs@chitosan and AgNPs@chitosan-NaF nanocomposites. The crystalline structure of the silver nanoparticles and their interaction with the chitosan matrix may influence the overall physiochemical properties of the nanocomposites (Govindan et al., 2012; Zhang et al., 2016; Chicea et al., 2024). Further characterization studies are needed to elucidate the precise relationship between the nanocomposite structure and their biological activities.

Of interest, the AgNPs@chitosan-NaF nanocomposites exhibited selective cytotoxicity toward cancer cells, with lower toxicity observed against normal lung fibroblast WI-38 cells compared to lung carcinoma epithelial A549 cells. These findings suggest that AgNPs@chitosan-NaF may have potential as targeted anticancer agents. However, further *in vivo* studies are needed to validate their efficacy and safety. *In vitro* cytotoxicity studies have limitations in fully recapitulating the complex biological environment in vivo, including the extracellular matrix and the immune system response. Therefore, preclinical studies are essential to assess the pharmacokinetics, biodistribution, and potential toxicity of AgNPs@chitosan-NaF in animal models. By addressing these limitations, we can gain a more comprehensive understanding of the therapeutic potential of these nanocomposites for cancer treatment beside *Klebsiella* treatment.

It was observed that the high dosage of AgNPs@chitosan did not exhibit any cytotoxic effect over a 24 h. This lack of cytotoxicity may be attributed to the gradual release of silver ions from the gel matrix. This discovery suggests that chitosan may be used as a capping ingredient in nanoparticles due to its biocompatibility (AshaRani et al., 2009). Our results are comparable to the findings reported in previous studies (Freire et al., 2016; Wongpreecha et al., 2018). The observed cytotoxicity of AgNPs@chitosan coated with NaF at varying doses may be attributed to the induction of reactive oxygen species by the presence of AgNPs, which is well recognized as a significant contributor to DNA damage. The genotoxicity resulting from the action of reactive oxygen species has been previously shown in the case of metal oxide nanoparticles and AgNPs that are capped with starch (AshaRani et al., 2009; Yang et al., 2009). The potential for further harm arises from the interaction between silver ions and DNA, resulting in alterations to the structure of DNA. Therefore, the findings of this study suggest that AgNPs@chitosan do not exhibit cytotoxic or genotoxic properties on normal cells. However, it is seen that these nanoparticles induce toxic effects when administered at concentrations beyond a certain threshold. The microscopic examination of WI-38 and A549 ATCC-185 cells treated with a bactericidal dosage of AgNPs@chitosan did not reveal any discernible alterations in comparison to the control cells. However, exposure to a high dose (up to 500 μg mL^−1^) resulted in anomalous cellular shape and a limited number of cellular extensions. This phenomenon may be attributed to disruptions in cytoskeletal functionality induced by the administration of nanoparticles.

The prevalence of *Klebsiella* colonization has been reported to vary widely across different populations, ranging from 5 to 87.7% (Shu et al., 2009; Shon et al., 2013; Zheng et al., 2018). In the present study, the prevalence of *Klebsiella* species among all obtained samples was determined to be 12.5% (15/120), with all isolates identified as *K. pneumoniae* through genotypic assay.

The *Klebsiella* prevalence finding is lower than that of Al Bshabshe et al. (2020) who reported 39%, but higher than a previous study at 9.9% (Ripabelli et al., 2018). The higher incidence of *K. pneumoniae* in our study highlights the importance of ongoing monitoring and implantation of infection control strategies to address the spread of this pathogen. The increased prevalence of *K. pneumoniae* in our study compared to earlier studies could be attributed to various factors, such as differences in study populations, healthcare facilities, or geographic locations. Additionally, the higher percentage of *Klebsiella* infections found in recent research may be due to the use of more sensitive diagnostic techniques. Compared to other common pathogens in these infected cases, *K. pneumoniae* is a significant concern due to its ability to form biofilms and produce extended-spectrum beta-lactamases (ESBLs), which confer resistance to many antibiotics (Vuotto et al., 2014; Costa, 2019; Ebrahem et al., 2024). Therefore, the relatively high prevalence of *Klebsiella pneumoniae* in our study underscores the need for effective infection prevention and control strategies.

These risk factors analysis that certain patient populations and sample types are at an increased risk for *Klebsiella* colonization. This highlights the need for targeted infection control measures, such as catheter care, hand hygiene, antibiotic stewardship, environmental disinfection, and surveillance, to reduce the burden of *Klebsiella* infections.

These findings of biofilm quantification suggest that different strains or clones of *Klebsiella pneumoniae* may have distinct biofilm-forming capacities (Zheng et al., 2018). Biofilm formation is often associated with increased antibiotic resistance and can complicate the treatment of infections. Furthermore, the ability of *K. pneumoniae* to form biofilms can contribute to the persistence of infections and the spread of the bacteria in healthcare settings (Guerra et al., 2022). Effective infection prevention and control measures, including proper hand hygiene, disinfection of surfaces, and antibiotic stewardship, are essential to address the challenge of *Klebsiella pneumoniae* biofilms.

The emergence of multidrug-resistant *Klebsiella* strains highlights the urgent need for effective antimicrobial strategies. In our study, a concerning 87% (n = 13) of isolates were classified as extreme drug-resistant (XDR, resistant to all classes of antimicrobial agents except 2 or fewer), and 13% were pan-resistant (resistant to all antimicrobial agents). Infections caused by these MDR strains are often difficult to treat, leading to prolonged illness, increased mortality, and higher healthcare costs. The limited availability of effective antibiotics can also contribute to the spread of MDR strains, making it challenging to control outbreaks (El Damaty et al., 2023; El-Demerdash et al., 2023c; El-Demerdash et al., 2023b; Megahed et al., 2023). Previous studies have reported similar or even higher rates of MDR *K. pneumoniae* in various regions worldwide (Moradigaravand et al., 2017; Huynh et al., 2020; Miftode et al., 2021; El-Demerdash et al., 2024). These findings underscore the global nature of this public health crisis. To address the challenge of MDR *K. pneumoniae*, it is essential to implement comprehensive infection prevention and control measures, promote the appropriate use of antibiotics, and support the development of new antimicrobial agents.

The agar well diffusion and microdilution results align with previous studies on the antibacterial properties of silver nanoparticles (Bruna et al., 2021; More et al., 2023) and suggest that the incorporation of sodium fluoride may enhance their efficacy. The antibacterial activity of AgNPs is likely due to the release of silver ions, the generation of reactive oxygen species, and physical disruption of bacterial cell components (Ahmad et al., 2017; Tripathi and Goshisht, 2022). The addition of chitosan and sodium fluoride may enhance the antibacterial activity of AgNPs and the generation of ROS through synergistic effects, improved stability, and enhanced penetration into bacterial cells.

The expression of biofilm-associated genes, including *treC*, *fimA*, *mrkA*, and *ecpA*, was investigated to elucidate the mechanisms underlying biofilm formation by *Klebsiella pneumoniae* isolates. The *treC* gene, involved in capsular polysaccharide production, and the *fimA* gene, encoding the major subunit of type 1 fimbriae, are key factors in biofilm initiation and development (Li and Ni, 2023). The *mrkA* gene has been implicated in the rapid formation of biofilms in *Klebsiella pneumoniae* (Murphy and Clegg, 2012). While the *ecpA* gene is associated with virulence and biofilm formation in other bacterial species (Alcántar-Curiel et al., 2013), its role in *Klebsiella* biofilm development requires further investigation.

The downregulation of these genes suggests that the nanocomposites may interfere with the molecular mechanisms underlying biofilm formation such as quorum sensing, extracellular matrix production, bacterial adhesion, and bacterial metabolism (Afrasiabi and Partoazar, 2024). These findings align with previous studies highlighting the importance of targeting biofilm-associated genes for developing effective antibiofilm strategies (Nadar et al., 2022).

## Conclusion

This study investigated the prevalence, antimicrobial susceptibility patterns, and biofilm formation of *Klebsiella pneumoniae* isolates, highlighting a significant public health concern due to the emergence of multidrug-resistant (MDR) and extensively drug-resistant (XDR) strains. To address this pressing issue, we developed chitosan-based silver nanoparticles (AgNPs) functionalized with sodium fluoride (AgNPs@chitosan-NaF).

The synthesized nanocomposites demonstrated promising antibacterial and antibiofilm activity against *K. pneumoniae in vitro*. However, further *in vivo* studies are necessary to validate their efficacy and safety in complex biological environments. Additionally, while the nanocomposites exhibited minimal cytotoxicity at lower concentrations, their potential toxicity at higher doses requires careful consideration and optimization of dosing strategies for clinical applications.

To gain a deeper understanding of the mechanisms of action, a more comprehensive molecular analysis, beyond biofilm-associated genes, is warranted. Nevertheless, these preliminary findings suggest that AgNPs@chitosan-NaF hold promise as a novel therapeutic approach for combating *K. pneumoniae* infections. Future research should focus on addressing these limitations and exploring the full potential of these nanocomposites.

## Data Availability

DNA sequence generated in this study was submitted to GenBank and assigned the accession number of PQ516970.

## References

[ref1] Abbas R. Chakkour M. Zein El Dine H. Obaseki E. F. Obeid S. T. Jezzini A.. (2024). General overview of Klebsiella pneumonia: epidemiology and the role of Siderophores in its pathogenicity. Biology (Basel) 13:78. doi: 10.3390/biology13020078, PMID: 38392297 PMC10886558

[ref2] Abd El-Emam M. M. El-Demerdash A. S. Abdo S. A. Abd-Elfatah E. B. El-Sayed M. M. Qelliny M. R.. (2024). The ameliorative role of *Aloe vera*-loaded chitosan nanoparticles on *Staphylococcus aureus* induced acute lung injury: targeting TLR/NF-$κ$B signaling pathways. Open Vet J 14, 416–427. doi: 10.5455/OVJ.2024.v14.i1.38, PMID: 38633182 PMC11018431

[ref3] Afrasiabi S. Partoazar A. (2024). Targeting bacterial biofilm-related genes with nanoparticle-based strategies. Front. Microbiol. 15:1387114. doi: 10.3389/fmicb.2024.1387114, PMID: 38841057 PMC11150612

[ref4] Ahmad A. Wei Y. Syed F. Tahir K. Rehman A. U. Khan A.. (2017). The effects of bacteria-nanoparticles interface on the antibacterial activity of green synthesized silver nanoparticles. Microb. Pathog. 102, 133–142. doi: 10.1016/j.micpath.2016.11.030, PMID: 27916692

[ref5] Al Bshabshe A. Al-Hakami A. Alshehri B. Al-Shahrani K. A. Alshehri A. A. Al Shahrani M. B.. (2020). Rising *Klebsiella pneumoniae* infections and its expanding drug resistance in the intensive care unit of a tertiary healthcare hospital, Saudi Arabia. Cureus 12:10060. doi: 10.7759/cureus.10060 PMC7520404 32999783

[ref6] Alcántar-Curiel M. D. Blackburn D. Saldaña Z. Gayosso-Vázquez C. Iovine N. la Cruz M. A.. (2013). Multi-functional analysis of *Klebsiella pneumoniae* fimbrial types in adherence and biofilm formation. Virulence 4, 129–138. doi: 10.4161/viru.22974, PMID: 23302788 PMC3654611

[ref7] Ali N. M. Mohamed G. A. E. El-Demerdash A. S. (2023). Impact of oral administration of Chitosan--nanoparticles on oxidative stress index and gut microbiota of heat stressed broilers. J Adv Vet Res 13, 997–1003.

[ref8] AshaRani P. V. Low G. Hande M. Valiyaveettil S. (2009). Cytotoxicity and genotoxicity of silver nanoparticles in human cells. ACS Nano 3, 279–290. doi: 10.1021/nn800596w, PMID: 19236062

[ref9] Bauer A. W. Kirby W. M. Sherris J. C. Turck M. (1966). Antibiotic susceptibility testing by a standardized single disk method. Am. J. Clin. Pathol. 45, 493–496. doi: 10.1093/ajcp/45.4_ts.493, PMID: 5325707

[ref10] Bruna T. Maldonado-Bravo F. Jara P. Caro N. (2021). Silver nanoparticles and their antibacterial applications. Int. J. Mol. Sci. 22:7202. doi: 10.3390/ijms22137202, PMID: 34281254 PMC8268496

[ref11] Chicea D. Nicolae-Maranciuc A. Chicea L.-M. (2024). Silver nanoparticles-chitosan nanocomposites: a comparative study regarding different chemical syntheses procedures and their antibacterial effect. Materials 17:1113. doi: 10.3390/ma17051113, PMID: 38473584 PMC10934116

[ref12] CLSI (2020). CLSI M100-ED29: 2021 performance standards for antimicrobial susceptibility testing. 30th Edn. Philadelphia, PA: CLSI, 50–51.

[ref13] Costa D. T. D. (2019). Virulence determinants and antibiotic resistance of extended-Spectrum Beta-lactamase (ESBL) producing *Klebsiella pneumoniae* isolated from hospital environment. Dhaka: Brac University.

[ref14] Desai N. Rana D. Salave S. Gupta R. Patel P. Karunakaran B.. (2023). Chitosan: a potential biopolymer in drug delivery and biomedical applications. Pharmaceutics 15:1313. doi: 10.3390/pharmaceutics15041313, PMID: 37111795 PMC10144389

[ref15] Ebrahem A. F. El-Demerdash A. S. Orady R. M. Nabil N. M. (2024). Modulatory effect of competitive exclusion on the transmission of ESBL *E. coli* in chickens. Probiotics Antimicrob. Proteins 16, 1087–1098. doi: 10.1007/s12602-023-10095-1, PMID: 37277569 PMC11126521

[ref16] El Damaty H. M. El-Demerdash A. S. Abd El-Aziz N. K. Yousef S. G. Hefny A. A. Abo Remela E. M.. (2023). Molecular characterization and antimicrobial susceptibilities of *Corynebacterium pseudotuberculosis* isolated from Caseous lymphadenitis of smallholder sheep and goats. Animals 13:2337. doi: 10.3390/ani13142337, PMID: 37508114 PMC10376069

[ref17] El-Demerdash A. S. Aggour M. G. El-Azzouny M. M. Abou-Khadra S. H. (2018). Molecular analysis of integron gene cassette arrays associated multi-drug resistant Enterobacteriaceae isolates from poultry. Cell. Mol. Biol. 64, 149–156. doi: 10.14715/cmb/2018.64.5.25, PMID: 29729709

[ref18] El-Demerdash A. S. Al Atfeehy N. M. Hamed R. I. Bakry N. R. Matter A. A. Eid S. (2023a). Mobile Colistin resistance determinants among Enterobacteriaceae isolated from different poultry species. J. Adv. Vet. Res. 13, 1004–1010.

[ref19] El-Demerdash A. S. Alfaraj R. Fared F. Saleh A. Dawwam G. E.. (2024). Essential oils as capsule disruptors: enhancing antibiotic efficacy against multidrug-resistant *Klebsiella pneumoniae*. Front. Microbiol. 15:1467460. doi: 10.3389/fmicb.2024.1467460, PMID: 39282565 PMC11392748

[ref20] El-Demerdash A. S. Mohamady S. N. Megahed H. M. Ali N. M. (2023b). Evaluation of gene expression related to immunity, apoptosis, and gut integrity that underlies Artemisia’s therapeutic effects in necrotic enteritis-challenged broilers. 3 Biotech 13:181. doi: 10.1007/s13205-023-03560-9, PMID: 37193331 PMC10182211

[ref21] El-Demerdash A. S. Mowafy R. E. Fahmy H. A. Matter A. A. Samir M. (2023c). Pathognomonic features of *Pasteurella multocida* isolates among various avian species in Sharkia governorate, Egypt. World J. Microbiol. Biotechnol. 39:335. doi: 10.1007/s11274-023-03774-2, PMID: 37807011 PMC10560635

[ref22] El-Demerdash A. S. Orady R. M. Matter A. A. Ebrahem A. F. (2023d). An alternative approach using Nano-garlic emulsion and its synergy with antibiotics for controlling biofilm-producing multidrug-resistant *Salmonella* in chicken. Indian J. Microbiol. 63, 632–644. doi: 10.1007/s12088-023-01124-2, PMID: 38034905 PMC10682320

[ref23] El-Naggar N. E.-A. Shiha A. M. Mahrous H. Mohammed A. B. A. (2022). Green synthesis of chitosan nanoparticles, optimization, characterization and antibacterial efficacy against multi drug resistant biofilm-forming *Acinetobacter baumannii*. Sci. Rep. 12:19869. doi: 10.1038/s41598-022-24303-5, PMID: 36400832 PMC9674591

[ref24] Franci G. Falanga A. Galdiero S. Palomba L. Rai M. Morelli G.. (2015). Silver nanoparticles as potential antibacterial agents. Molecules 20, 8856–8874. doi: 10.3390/molecules20058856, PMID: 25993417 PMC6272636

[ref25] Freire P. L. L. Albuquerque A. J. R. Farias I. A. P. da Silva T. G. Aguiar J. S. Galembeck A.. (2016). Antimicrobial and cytotoxicity evaluation of colloidal chitosan--silver nanoparticles--fluoride nanocomposites. Int. J. Biol. Macromol. 93, 896–903. doi: 10.1016/j.ijbiomac.2016.09.052, PMID: 27642129

[ref26] Garcia L. S. (2010). Clinical microbiology procedures handbook. Washington, DC: American Society for Microbiology Press.

[ref27] Govindan S. Nivethaa E. A. K. Saravanan R. Narayanan V. Stephen A. (2012). Synthesis and characterization of chitosan--silver nanocomposite. Appl. Nanosci. 2, 299–303. doi: 10.1007/s13204-012-0109-5

[ref28] Guerra M. E. S. Destro G. Vieira B. Lima A. S. Ferraz L. F. C. Hakansson A. P.. (2022). *Klebsiella pneumoniae* biofilms and their role in disease pathogenesis. Front. Cell. Infect. Microbiol. 12:877995. doi: 10.3389/fcimb.2022.877995, PMID: 35646720 PMC9132050

[ref29] Hao S. Wang B. Wang Y. Zhu L. Wang B. Guo T. (2013). Preparation of Eudragit L 100-55 enteric nanoparticles by a novel emulsion diffusion method. Colloids Surf. B Biointerfaces 108, 127–133. doi: 10.1016/j.colsurfb.2013.02.036, PMID: 23537830

[ref30] Hashem N. M. Essawi W. M. El-Demerdash A. S. El-Raghi A. A. (2024). Biomolecule-producing probiotic bacterium *Lactococcus lactis* in free or Nanoencapsulated form for endometritis treatment and fertility improvement in buffaloes. J. Funct. Biomater. 15:138. doi: 10.3390/jfb15060138, PMID: 38921512 PMC11204555

[ref31] Huynh B.-T. Passet V. Rakotondrasoa A. Diallo T. Kerleguer A. Hennart M.. (2020). *Klebsiella pneumoniae* carriage in low-income countries: antimicrobial resistance, genomic diversity and risk factors. Gut Microbes 11, 1287–1299. doi: 10.1080/19490976.2020.1748257, PMID: 32404021 PMC7527070

[ref32] Ibrahim M. E. (2023). Risk factors in acquiring multidrug-resistant *Klebsiella pneumoniae* infections in a hospital setting in Saudi Arabia. Sci. Rep. 13:11626. doi: 10.1038/s41598-023-38871-7, PMID: 37468757 PMC10356761

[ref33] Kaur P. Choudhary A. Thakur R. (2013). Synthesis of chitosan-silver nanocomposites and their antibacterial activity. Int. J. Sci. Eng. Res. 4:869.

[ref34] Larm N. E. Thon J. A. Vazmitsel Y. Atwood J. L. Baker G. A. (2019). Borohydride stabilized gold--silver bimetallic nanocatalysts for highly efficient 4-nitrophenol reduction. Nanoscale Adv. 1, 4665–4668. doi: 10.1039/C9NA00645A, PMID: 36133135 PMC9418733

[ref35] Li Y. Ni M. (2023). Regulation of biofilm formation in *Klebsiella pneumoniae*. Front. Microbiol. 14:1238482. doi: 10.3389/fmicb.2023.1238482, PMID: 37744914 PMC10513181

[ref36] Mba I. E. Nweze E. I. (2021). Nanoparticles as therapeutic options for treating multidrug-resistant bacteria: research progress, challenges, and prospects. World J. Microbiol. Biotechnol. 37, 1–30. doi: 10.1007/s11274-021-03070-x, PMID: 34046779 PMC8159659

[ref37] Megahed M. M. M. El-Nagar A. M. A. El-Demerdash A. S. Ayoub M. A. Tolba H. M. N. (2023). Evaluation and development of diagnostic tools for rapid detection of *Riemerella anatipestifer* and *Pasteurella multocida* in ducks. J. Adv. Vet. Anim. Res. 10, 211–221. doi: 10.5455/javar.2023.j671, PMID: 37534083 PMC10390669

[ref38] Mehr F. P. Khanjani M. Vatani P. (2015). Synthesis of nano-ag particles using sodium borohydride. Orient. J. Chem. 31, 1831–1833. doi: 10.13005/ojc/310367

[ref39] Miftode I. L. Nastase E. V. Miftode R. S. Iancu L. Luncă C. Păduraru D. T.. (2021). Insights into multidrug-resistant *K. pneumoniae* urinary tract infections: from susceptibility to mortality. Exp. Ther. Med. 22:1086. doi: 10.3892/etm.2021.10520, PMID: 34447478 PMC8355719

[ref40] Moradigaravand D. Martin V. Peacock S. J. Parkhill J. (2017). Evolution and epidemiology of multidrug-resistant *Klebsiella pneumoniae* in the United Kingdom and Ireland. MBio 8, 10–1128. doi: 10.1128/mBio.01976-16, PMID: 28223459 PMC5358916

[ref41] More P. R. Pandit S. De Filippis A. Franci G. Mijakovic I. Galdiero M. (2023). Silver nanoparticles: bactericidal and mechanistic approach against drug resistant pathogens. Microorganisms 11:369. doi: 10.3390/microorganisms11020369, PMID: 36838334 PMC9961011

[ref42] Murphy C. N. Clegg S. (2012). *Klebsiella pneumoniae* and type 3 fimbriae: nosocomial infection, regulation and biofilm formation. Future Microbiol. 7, 991–1002. doi: 10.2217/fmb.12.74, PMID: 22913357

[ref43] Nadar S. Khan T. Patching S. G. Omri A. (2022). Development of antibiofilm therapeutics strategies to overcome antimicrobial drug resistance. Microorganisms 10:303. doi: 10.3390/microorganisms10020303, PMID: 35208758 PMC8879831

[ref44] Pawar O. Deshpande N. Dagade S. Waghmode S. Nigam Joshi P. (2016). Green synthesis of silver nanoparticles from purple acid phosphatase apoenzyme isolated from a new source *Limonia acidissima*. J. Exp. Nanosci. 11, 28–37. doi: 10.1080/17458080.2015.1025300

[ref45] Razali N. M. Wah Y. B. (2011). Power comparisons of shapiro-wilk, kolmogorov-smirnov, lilliefors and anderson-darling tests. J. Stat. Model. Anal. 2, 21–33.

[ref46] Ripabelli G. Tamburro M. Guerrizio G. Fanelli I. Flocco R. Scutellà M.. (2018). Tracking multidrug-resistant *Klebsiella pneumoniae* from an Italian hospital: molecular epidemiology and surveillance by PFGE, RAPD and PCR-based resistance genes prevalence. Curr. Microbiol. 75, 977–987. doi: 10.1007/s00284-018-1475-3, PMID: 29523910

[ref47] Rudramurthy G. R. Swamy M. K. Sinniah U. R. Ghasemzadeh A. (2016). Nanoparticles: alternatives against drug-resistant pathogenic microbes. Molecules 21:836. doi: 10.3390/molecules21070836, PMID: 27355939 PMC6273897

[ref48] Saad M. F. Elsayed M. M. Khder M. Abdelaziz A. S. El-Demerdash A. S. (2024). Biocontrol of multidrug resistant pathogens isolated from fish farms using silver nanoparticles combined with hydrogen peroxide insight to its modulatory effect. Sci. Rep. 14:7971. doi: 10.1038/s41598-024-58349-4, PMID: 38575637 PMC10994946

[ref49] SAS (2012). SAS/OR 9.3 User’s guide: Mathematical programming examples, SAS/STAT statistics. North Carolina, CA: SAS Institute.

[ref50] Sharaf M. Sewid A. H. Hamouda H. I. Elharrif M. G. El-Demerdash A. S. Alharthi A.. (2022). Rhamnolipid-coated Iron oxide nanoparticles as a novel multitarget candidate against major Foodborne *E. coli* serotypes and methicillin-Resistant *S. aureus*. Microbiol. Spectr. 10, e00250–e00222. doi: 10.1128/spectrum.00250-22 35852338 PMC9430161

[ref51] Shon A. S. Bajwa R. P. S. Russo T. A. (2013). Hypervirulent (hypermucoviscous) *Klebsiella pneumoniae*: a new and dangerous breed. Virulence 4, 107–118. doi: 10.4161/viru.22718, PMID: 23302790 PMC3654609

[ref52] Shu H.-Y. Fung C.-P. Liu Y.-M. Wu K.-M. Chen Y.-T. Li L.-H.. (2009). Genetic diversity of capsular polysaccharide biosynthesis in *Klebsiella pneumoniae* clinical isolates. Microbiology (N Y) 155, 4170–4183. doi: 10.1099/mic.0.029017-0, PMID: 19744990

[ref53] Steel R. G. D. Torrie J. H. (1980). Principles and procedures of statistics. New York: McGraw-Hill Publishing Company, 481.

[ref54] Stepanović S. Vuković D. Dakić I. Savić B. Švabić-Vlahović M. (2000). A modified microtiter-plate test for quantification of staphylococcal biofilm formation. J. Microbiol. Methods 40, 175–179. doi: 10.1016/S0167-7012(00)00122-6, PMID: 10699673

[ref55] Tambekar D. H. Dhanorkar D. V. Gulhane S. R. Khandelwal V. K. Dudhane M. N. (2006). Antibacterial susceptibility of some urinary tract pathogens to commonly used antibiotics. Afr. J. Biotechnol. 5:17.

[ref56] Tripathi N. Goshisht M. K. (2022). Recent advances and mechanistic insights into antibacterial activity, antibiofilm activity, and cytotoxicity of silver nanoparticles. ACS Appl. Biomater 5, 1391–1463. doi: 10.1021/acsabm.2c00014, PMID: 35358388

[ref57] Turton J. F. Perry C. Elgohari S. Hampton C. V. (2010). PCR characterization and typing of *Klebsiella pneumoniae* using capsular type-specific, variable number tandem repeat and virulence gene targets. J. Med. Microbiol. 59, 541–547. doi: 10.1099/jmm.0.015198-0, PMID: 20110386

[ref58] Venkataraju J. L. Sharath R. Chandraprabha M. N. Neelufar E. Hazra A. Patra M. (2014). Synthesis, characterization and evaluation of antimicrobial activity of zinc oxide nanoparticles. J. Biochem. Technol. 3, 151–154.

[ref59] Vuotto C. Longo F. Balice M. P. Donelli G. Varaldo P. E. (2014). Antibiotic resistance related to biofilm formation in *Klebsiella pneumoniae*. Pathogens 3, 743–758. doi: 10.3390/pathogens3030743, PMID: 25438022 PMC4243439

[ref60] Wongpreecha J. Polpanich D. Suteewong T. Kaewsaneha C. Tangboriboonrat P. (2018). One-pot, large-scale green synthesis of silver nanoparticles-chitosan with enhanced antibacterial activity and low cytotoxicity. Carbohydr. Polym. 199, 641–648. doi: 10.1016/j.carbpol.2018.07.039, PMID: 30143172

[ref61] Wu M.-C. Lin T.-L. Hsieh P.-F. Yang H.-C. Wang J.-T. (2011). Isolation of genes involved in biofilm formation of a *Klebsiella pneumoniae* strain causing pyogenic liver abscess. PLoS One 6:e23500. doi: 10.1371/journal.pone.0023500, PMID: 21858144 PMC3155550

[ref62] Yang H. Liu C. Yang D. Zhang H. Xi Z. (2009). Comparative study of cytotoxicity, oxidative stress and genotoxicity induced by four typical nanomaterials: the role of particle size, shape and composition. J. Appl. Toxicol. 29, 69–78. doi: 10.1002/jat.1385, PMID: 18756589

[ref63] Yuan J. S. Reed A. Chen F. Stewart C. N. (2006). Statistical analysis of real-time PCR data. BMC Bioinformatics 7:85. doi: 10.1186/1471-2105-7-85, PMID: 16504059 PMC1395339

[ref64] Zhang X.-F. Liu Z.-G. Shen W. Gurunathan S. (2016). Silver nanoparticles: synthesis, characterization, properties, applications, and therapeutic approaches. Int. J. Mol. Sci. 17:1534. doi: 10.3390/ijms17091534, PMID: 27649147 PMC5037809

[ref65] Zheng J. Lin Z. Chen C. Chen Z. Lin F. Wu Y.. (2018). Biofilm formation in *Klebsiella pneumoniae* bacteremia strains was found to be associated with CC23 and the presence of wcaG. Front. Cell. Infect. Microbiol. 8:21. doi: 10.3389/fcimb.2018.00021, PMID: 29527517 PMC5829044

